# Short QT intervals in African lions

**DOI:** 10.1113/EP092203

**Published:** 2024-10-10

**Authors:** Frederik S. Scharling, Ditte‐Mari Sandgreen, Julia Stagegaard, Vibeke S. Elbrønd, Stefano Vincenti, Jonas L. Isaksen, Tobias Wang, Rory P. Wilson, Richard Gunner, Nikki Marks, Stephen H. Bell, Martin C. van Rooyen, Nigel C. Bennett, Daniel W. Hart, Angela C. Daly, Mads F. Bertelsen, D. Michael Scantlebury, Kirstine Calloe, Morten B. Thomsen

**Affiliations:** ^1^ Department of Veterinary and Animal Sciences University of Copenhagen Frederiksberg Denmark; ^2^ Givskud Zoo – Zootopia Give Denmark; ^3^ Ree Park – Safari Ebeltoft Denmark; ^4^ Department of Biomedical Sciences University of Copenhagen Copenhagen Denmark; ^5^ Zoophysiology, Department of Biology Aarhus University Aarhus Denmark; ^6^ Department of Biosciences University of Swansea Swansea UK; ^7^ Max Planck Institute of Animal Behavior Konstanz Germany; ^8^ School of Biological Sciences Queen's University Belfast Belfast UK; ^9^ Department of Zoology and Entomology University of Pretoria Pretoria South Africa; ^10^ Veterinary Wildlife Services, Conservation Services Department South African National Parks Pretoria South Africa; ^11^ Center for Zoo and Wild Animal Health Copenhagen Zoo Copenhagen Denmark

**Keywords:** circadian, feline, *Panthera leo*, predator, Purkinje, QT

## Abstract

The cardiac conduction system in large carnivores, such as the African lion (*Panthera leo*), represents a significant knowledge gap in both veterinary science and in cardiac electrophysiology. Short QT intervals have been reported from zoo‐kept, anaesthetized lions, and our goal was to record the first ECGs from wild, conscious lions roaming freely, and compare them to zoo‐kept lions under the hypothesis that short QT is unique to zoo‐kept lions. Macroscopic and histological examinations were performed on heart tissue removed from nine healthy zoo lions. ECGs were recorded from the nine anaesthetized zoo‐kept lions, and from 15 anaesthetized and conscious wild lions in Africa. Our histological and topographical description of the lion's heart matched what has previously been published. In conscious lions, the ECG recordings revealed a mean heart rate of 70 ± 4 beats/min, with faster heart rates during the night. PQ and QT intervals were heart rate dependent in the conscious lions. Interestingly, QT intervals recorded in wild lions were markedly longer than QT intervals from zoo lions (398 ± 40 vs. 297 ± 9 ms, respectively; *P *< 0.0001). Anaesthesia or heart rate did not account for this difference. We provide a comprehensive description of the cardiac anatomy and electrophysiology of wild and zoo‐kept lions. QT intervals were significantly shorter in zoo lions, suggesting functional disparities in cardiac electrophysiology between wild and zoo‐kept lions, potentially related to physical fitness. These findings underscore the plasticity of cardiac electrophysiology and may be of value when reintroducing endangered species into the wild and when managing lions in human care.

## Abstract (highlights)

- **What is the central question of this study?**
  How do electrocardiograms from zoo lions and free‐roaming wild lions compare?
- **What is the main finding and its importance?**
  QT intervals in zoo lions are shorter compared to wild lions, which may be relevant for our understanding of animals in human care and may influence conservation strategies.

## 1 INTRODUCTION

The lion (*Panthera leo*) is endemic to the continents of Africa and India and is amongst the largest predators on Earth. The conservation status of these charismatic animals is classed as *vulnerable*, the first of three categories of *threatened* species, according to the International Union for Conservation of Nature (Nicholson et al., 2023). Most wild lions live in familial prides on open grasslands, feeding on large mammals, and are mainly active during the night (Seyrling et al., 2022). Given the vulnerable status and decline of lions in the wild, zoo lions under human care are now an important source of genetic diversity.

Lions are a prominent feature in zoos and wildlife parks globally due to their iconic status and appeal. Managing the population of these zoo‐kept lions to enhance genetic diversity and prevent inbreeding is a well‐established practice. However, they are housed in smaller enclosures, relative to their wild counterparts, do not hunt and are more accustomed to the daytime routines associated with human care. Most scientific studies on lions have been made on zoo‐kept animals with limited comparison to populations in their natural habitat.

The topography and anatomy of the lion's heart was recently reviewed to support the increased demands for veterinary treatment of lions (Marais & Crole, 2022), and three studies have reported on the electrocardiogram of immobilized and anaesthetized zoo lions (Larsson et al., 2008; Omóbòwálé et al., 2017; Reilly et al., 2014). To our knowledge, the ECG from a wild lion has not been reported, and it therefore remains unknown whether electrophysiological data from zoo lions are representative of animals living in their natural habitat. Understanding the normal ECG patterns of conscious, wild lions in their natural habitat could provide a baseline for estimating responses to environmental changes and stress in response to human activities, and facilitate comparison to the physiology of lions bred in captivity.

In the current study, we aimed to provide an electrohistological and electrophysiological comparison between wild and zoo‐kept lions. Macroscopic dissection of the hearts of clinically healthy lions was performed along with thorough histological examinations of the conduction system, including the sinoatrial and atrioventricular nodes. In addition, we report and compare electrophysiological parameters from anaesthetized and conscious, wild lions, alongside detailed ECG recordings from zoo lions bred in captivity. We found that zoo‐kept lions have considerably and significantly shorter QT intervals than those of wild lions. To our knowledge, this study is the first to report electrocardiographic parameters from conscious, wild lions roaming in their natural habitat.

## 2 METHODS

### 2.1 Ethical approval

The collection of data during anaesthesia before euthanasia of the zoo lions was approved by the Danish Animal Experiments Inspectorate (license number 2017‐15‐0201‐01219). Ethical approval for the wild animal work was given by Animal Welfare Ethical Review Body at Queen's University Belfast (AWERB), approval number SU‐Ethics‐Student‐260919/1894, reference IP‐1819‐30. Conditions and approval for lion fieldwork were granted by Queen's University of Belfast (QUB‐BS‐AREC‐18‐006) and the University of Pretoria (NAS061‐19). A research permit and AUCC approval was provided by South African National Parks (Permit number SCAM 1550). All procedures conformed to the guidelines from Directive 2010/63/EU of the European Parliament on the protection of animals used for scientific purposes.

### 2.2 Animals

Nine African lions (*P. leo*), aged 1–8 years, bred and housed in Danish zoos were investigated in the present study. The recordings and tissue sampling were done opportunistically from animals euthanized as part of the population management in the zoos, independent of the current study (Bertelsen, 2018).

Electrocardiograms were recorded from two female lions from Copenhagen Zoo (Copenhagen, Denmark); two males and one female from Ree Park (Ebeltoft, Denmark); and four female lions from Givskud Zoo (Give, Denmark) in 2018 and 2019.

Anaesthesia was induced by a combination of ketamine (3 mg/kg), medetomidine (30 µg/kg), midazolam (0.2 mg/kg) and butorphanol (0.1 mg/kg). The body mass of the individual animal was gauged by experienced zoo staff, and anaesthesia mix was delivered by darting and direct intramuscular injection. Body mass was determined postmortem on six lions and ranged between 79 and 120 kg. The zoo lions were euthanized by sodium pentobarbital.

In addition, 24 h ECGs were obtained from 15 wild lions from the Kgalagadi Transfrontier Park (South Africa National Parks, South Africa) in 2019. These lions were estimated to be between 3 and 12 years of age and comprised nine females and six males.

The lions were anaesthetized by intramuscular darting with a mix of tiletamine and zolazepam (Zoletil, 1.15 mg/kg) and medetomidine (50 µg/kg) with additional direct intramuscular injection of ketamine (0.9–1.0 mg/kg), if prolonged anaesthesia was required. All animals received post‐surgical analgesia and antimicrobial prophylaxis with meloxicam (2.2 mg/kg) and ceftiofur (7.5 mg/kg), respectively. Body mass ranged between 99 and 215 kg. The wild animals were not euthanized after the experiments but were released back into the National Park.

### 2.3 Tissue sampling

Tissue samples were collected from the zoo lions. Histological samples were obtained from the area of the sinoatrial (SA) node, the atrioventricular (AV) node and the left ventricle. Care was taken not to damage the free‐running Purkinje fibres on the endocardial surface of the ventricle. Tissue samples were stored in 4% buffered formaldehyde and embedded in paraffin and cut into 2–4 µm‐thick sections and stained with resorcin–fuchsin. Resorcin–fuchsin stains collagen red, elastic fibres dark purple/black, muscle cells yellow‐greyish and the nuclei black. All histological preparations and procedures were performed at the Histology Core Facility at the Section of Pathobiological Sciences, Department of Veterinary and Animal Sciences, University of Copenhagen, Denmark. The morphology was documented using a Ricoh GX‐200 and a Canon Powershot G1X camera. Microscopy images were obtained using a DMR microscope (Leica Microsystems, Wetzlar, Germany) and LAS program (Leica Application Suite) V4.10, Las Core (Leica Microsystems).

### 2.4 Electrocardiograms

The anaesthetized zoo lions were positioned in right lateral recumbency. Custom‐made electrodes were used to obtain two ECGs: (i) a six‐lead Einthoven configuration, and (ii) a three‐lead orthogonal configuration. A ground electrode for both configurations was placed on the right hind limb above the stifle.

For the Einthoven configuration, a common negative lead electrode was placed at the caudal edge of the triceps muscle (5–10 cm dorsal to olecranon) on the right foreleg, with one positive lead placed at the same position on left front limb (lead I) and a second positive lead placed on the left hind limb above the stifle joint (lead II).

For the orthogonal configuration, the electrodes for the *X* lead were positioned on each side of the thorax between the third and the sixth rib, closest to where the heart was judged to be. The negative lead was placed on the right side of the animal. The electrodes for the *Y* lead were positioned on the left descending pectoralis muscle near the midline at the breast (negative) and the left hind limb above the stifle joint (positive). The electrodes for the *Z* lead were positioned ventrally at the level of the seventh sternebrae (negative) and dorsally at the level of the seventh thoracic vertebrae (positive).

All ECGs were measured during the daytime (range 08.30–13.30 h). The ECGs were amplified (ML138 Bio Amp from ADInstruments, Dunedin, New Zealand), digitized (PowerLab 16/30, ADInstruments) and recorded at 4 kHz (LabChart v8, ADInstruments) for no less than 2 min in each configuration.

### 2.5 Data loggers

Commercially available data loggers (DST centi‐HRT or milli‐HRT, Star‐Oddi, Garðabær, Iceland) were used to obtain ECGs from conscious, freely roaming, wild lions in South Africa. Fifteen lions from the Kgalagadi Transfrontier Park were anaesthetized as detailed above. Data loggers were implanted on the left side of the thorax at the level of the heart's base.

Lions were equipped with a LiteTrack GPS collar (Lotek Wireless, Newmarket ON, Canada) which enabled them to be located in the field (Gunner et al., 2023). The implanted data loggers were programmed to record heart rate every 2 min and to record a 3‐ or 7.5‐s ECG trace at 200 Hz every eighth minute. After 14 days, the lions were recaptured to explant the data loggers. Usable ECGs were obtained from 12 animals.

### 2.6 Analysis

Anatomical and histological samples were compared and described. ECGs from zoo lions were analysed in LabChart; whereas Mercury (v6.30; Star‐Oddi) was used for analysing ECGs from the data loggers.

We determined RR, PQ, QRS and QT intervals in each of the nine zoo lions using lead II in Einthoven's configuration. The two‐dimensional (2D) vector loops in the plane defined by the Einthoven leads were obtained by plotting lead aVF as a function of the perpendicular lead I. Three‐dimensional (3D) vector loop cardiograms were plotted based on leads *X*, *Y* and *Z* in the orthogonal configuration. The mean electrical axis (MEA) was defined as the longest vector in the 3D vectorcardiogram. The mean ± standard deviation is reported.

We determined the mean RR interval every hour for 24 consecutive hours >5 days after surgery, from the data from the conscious wild lions. Recordings from three lions were excluded because the quality of the ECGs was insufficient for reliable analysis. A 24 h cosinor function was fitted to the data, as described previously (Gottlieb et al., 2021). We identified periods with a heart rate of 30, 40, 50, 60, 70, 80, 90, 100 and 120 beats/min, where PQ and QT intervals were determined. This analysis was performed twice by two different researchers, and the outcome was comparable with little intra‐observer variance (not shown).

Finally, we analysed the very first few ECG epochs with clear ECG morphologies. These ECGs were recorded when the lions were fully anaesthetized. We then compared these ECGs with ECGs obtained from the same, freely roaming animal >5 days after surgery at identical heart rates. For this comparison, we used a paired Student's *t*‐test. The ECG parameters from anaesthetized, wild lions were compared to the ones obtained from the zoo lions using an unpaired Student's *t*‐test.

## 3 RESULTS

**FIGURE 1:**
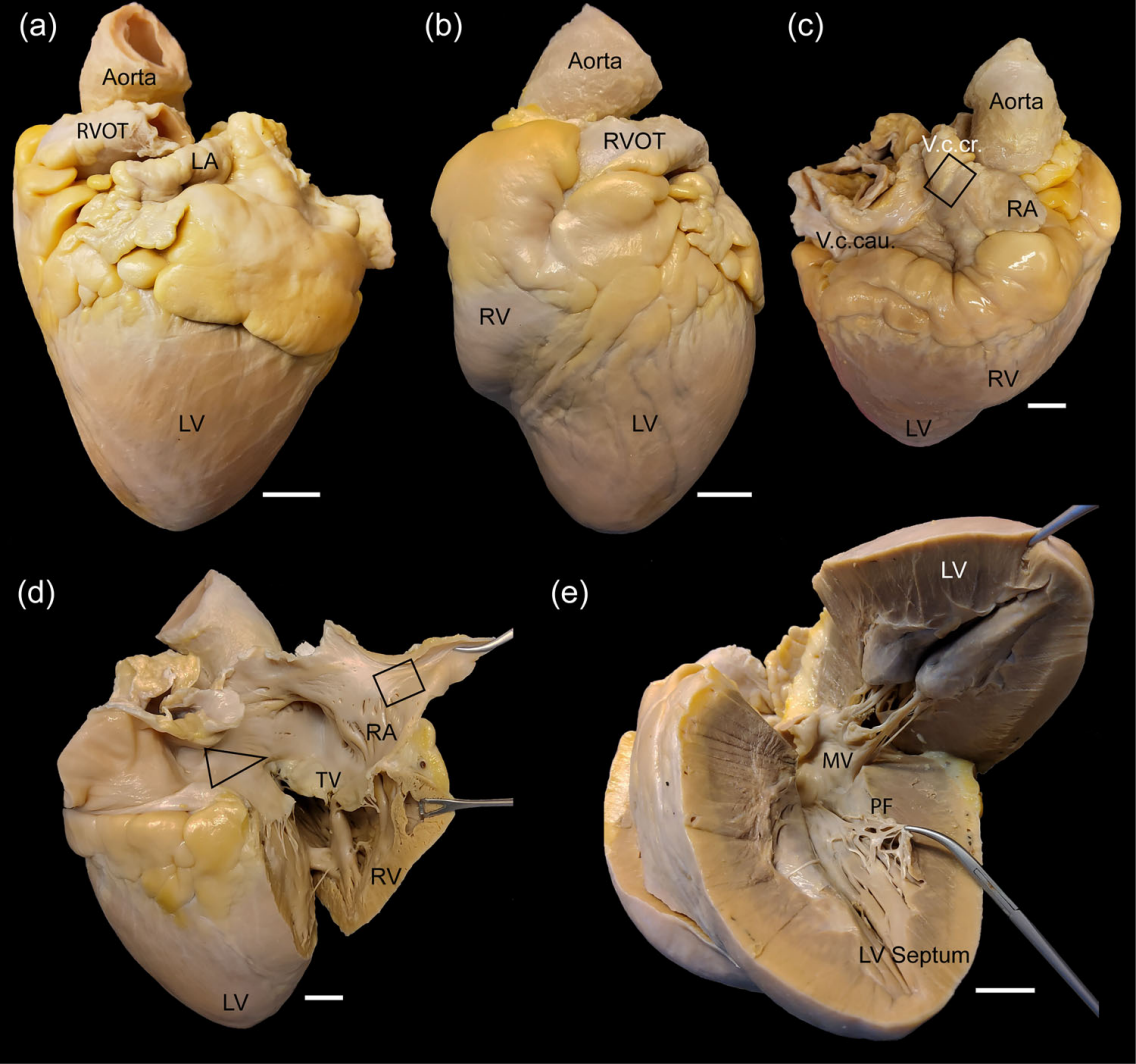
The lion heart. (a) A caudal view of the lion's heart. The left ventricle (LV), left auricle (LA), the right ventricular outflow tract (RVOT) and the aorta are indicated. Scale bar 1 cm. (b) Facies auricularis, a lateral view of the heart surface marking the tips of the auticulae atriorum, facing the left thoracic wall of the animal. Abbreviations as in (a); RV, right ventricle. Scale bar 1 cm. (c) An oblique basal view showing the cranial caval vein (V.c.cr) and the caudal caval vein (V.c.cau). The black square indicates the section where the sinoatrial node is located. Scale bar 1 cm. (d) The right atrium opened by an incision through the caval veins and the right ventricle by an incision following the septal wall. The triscupidal valve (TV) separates the right atrium and ventricle. The triangle of Koch is indicated by a black triangle. The atrioventricular node is located at the base of the triangle and the bundle of His toward the pointy end. The sinus‐node area is indicated by the black box. Scale bar 1 cm. (e) The left ventricle opened up by two insicions parallel to the septal wall. The mitral valve (MV) marks the transition from the left atrium to ventricle. A network of free‐running Purkinje strands or fibres (PF) is visible within the left ventricle. Scale bar 1 cm.

The heart is located in the thoracic cavity between the fourth and the sixth rib caudally to the forelimbs. The apex of the heart points toward the sterno‐diaphramatic junction and is supported in this position by the phrenicopericardial ligament. We confirm the detailed topographic description of the thorax and heart of the African lion from Marais and Crole (2022). Figure 1 shows different perspectives of a lion heart *ex vivo* with emphasis on the specialized conduction system.

### 3.1 Morphology the lion heart

Caudal (Figure 1a) and left lateral (Figure 1b) views show the external features of the lion's heart. The sinoatrial node is located at the junction between the right auricle and the cranial vena cava (Figure 1c, d, square). The atrioventricular node is positioned at the base of the right atrium (Figure 1d, triangle). The tricuspid valve separates the right atrium and ventricle (Figure 1d). In contrast, the left atrium and ventricle are separated by the mitral valves (Figure 1e). Close to the apex of the ventricle a large network of free‐running Purkinje fibres is observed (Figure 1e).

### 3.2 Anatomy and function of the sinoatrial node

**FIGURE 2:**
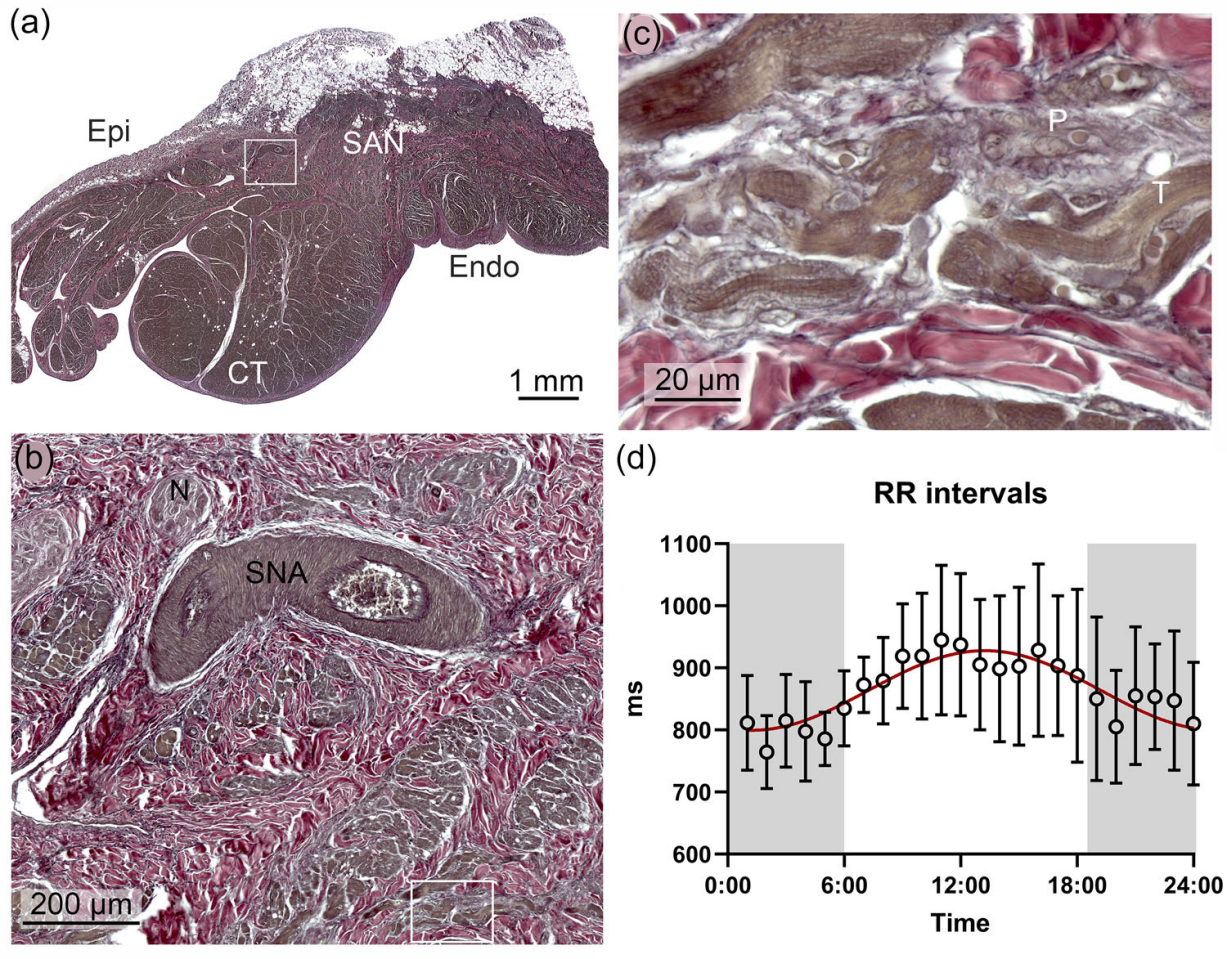
The sinoatrial node and RR intervals during a 24‐h cycle. (a) The sinoatrial node (SAN) is found sub‐epicardially (Epi) under the terminal crest (CT). Endo, endocardium. (b) A section corresponding to the white rectangle in (a), showing a branch of the central sinoatrial node artery (SNA) surrounded by extracellular matrix and islets of SAN cells and nerve fibres (N). (c) A section corresponding to the white rectangle in (b), showing pale, round pacemaker cells (P) as well as short, branching and striated transitional cells (T). (d) RR intervals during a 24‐h cycle from the ECG loggers placed in the freely roaming lions in Kgalagadi Transfrontier Park (*n* = 12). The red line illustrates the cosinor fit to the data. Error bars indicate the standard deviation. The dark period (18.30 h to 06.00 h) is indicated in grey.

The sinoatrial node is close to the epicardium, superior to the crista terminalis (Figure 2a) and around the central sinus‐node artery (Figure 2b). The nodal area is infiltrated by large irregularly organized collagen fibres, of which several present a wavy, undulating pattern, providing mechanical flexibility. A network of thin elastic fibres surrounds the collagen fibres. Islets of small, round pacemaker cells (P‐cells) intermingled with short branching transitional cells (T‐cells) are arranged in the connective tissue. The P‐cell cytoplasm appears pale, contains a few myofibrils, and has a centrally located nucleus. The T‐cells have cross‐striations and appear connected with the P‐cells (Figure 2b, c).

Heart rate is the functional readout of the SA node, so to describe the 24 h rhythm in heart rate, we analysed RR intervals on the ECGs recorded from the conscious, wild lions (Figure 2d). The average RR interval was 864 ± 52 ms (heart rate: 70 ± 4 beats/min; *n* = 12) with a clear 24 h rhythm, where the higher heart rate at night is consistent with the nocturnal activity of wild lions. The amplitude of the mathematically fitted 24 h rhythm in RR intervals was 128 ms from trough to peak (range 65–75 beats/min).

### 3.3 Anatomy and function of the atrioventricular node and ventricular conduction system

**FIGURE 3:**
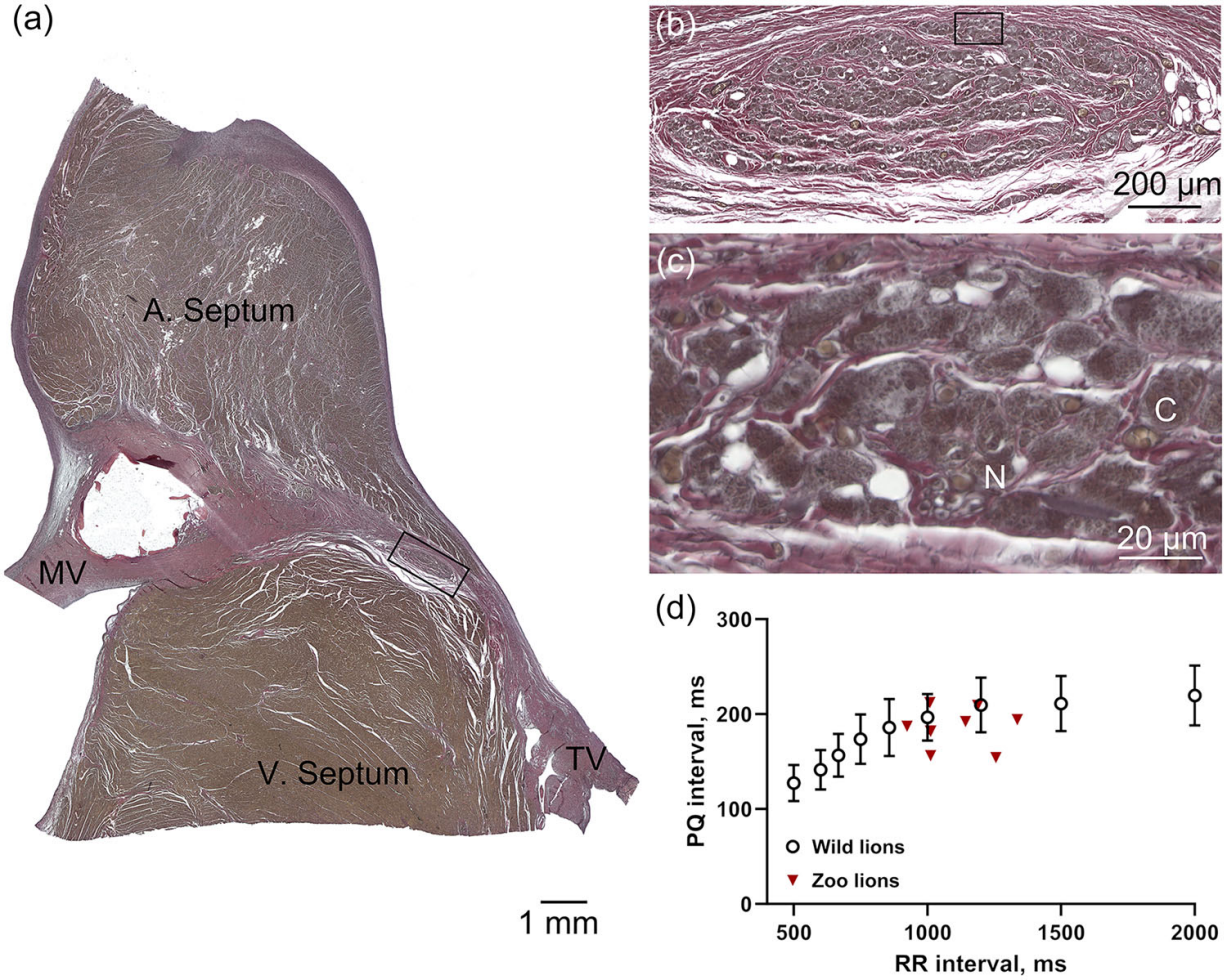
Atrioventricular node and PQ intervals. (a) The fibrous body is continuous with the mitral valve (MV) and the triscupidal valve (TV) and separates the atria from the ventricles. A. Septum, atrial septum; V. septum, ventricular septum. (b) A section corresponding to the black rectangle in (a), showing the penetrating bundle of His. (c) Enlargement of the area indicated by the rectangle in (b), showing Purkinje fibres (PF), nerves (N) and capillaries (C). (d) The PQ interval as a function of the RR interval (black symbols) from the ECG loggers placed in the freely roaming lions in Kgalagadi Transfrontier Park (*n* = 12). For comparison, the PQ interval as function of the RR interval from the individual anaesthetized zoo lions is plotted in red. Error bars indicate the standard deviation.

The atrioventricular (AV) node is located in the atrial septum near the right surface, close to the coronary sinus and the Eustachian valve (Figure 1d). Koch's triangle is defined by the ostium of coronary sinus, the tendon of Todaro, and the septal leaflet of the tricuspid valve. The triangle identifies the location of the AV node: at the apex of the triangle, opposite the coronary sinus, the AV node merges with the bundle of His at the central fibrous body of the septum. The bundle of His penetrates the fibrous body and electrically connects the atria and ventricles (Figure 3a and b). The bundle is vascularized and innervated, and the myocytes of the bundle of His resemble Purkinje fibres (Figure 3c).

The PQ interval on the ECG describes the time from SA node depolarization until the impulse has reached the bundle of His and is primarily determined by the delay of the impulse in the AV node. In the conscious, wild lions, the PQ interval varied between 127 ± 19 and 220 ± 32 ms when the heart rate decreased from 120 to 30 beats/min (RR interval prolonged from 500 to 2000 ms; Figure 3d). To compare the PQ intervals in the conscious, wild lions to the PQ intervals measured in anaesthetized zoo lions, we plotted the data on the same graph. No obvious differences were observed, and no statistical comparison was made.

**FIGURE 4:**
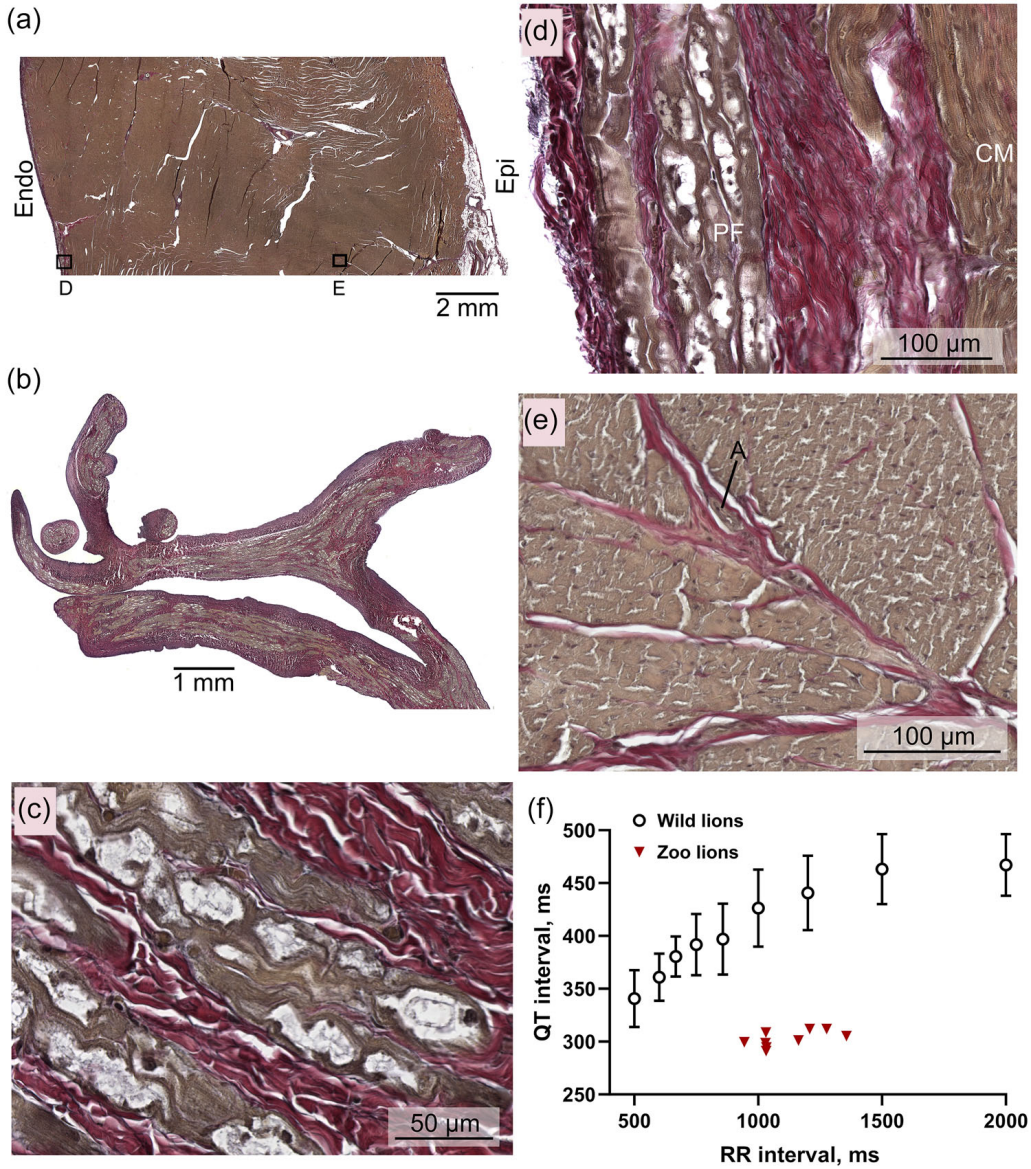
The left ventricle. (a) Transmural section of a lion left ventricle. On the epicardium (Epi), fat cells and several arteries can be observed. Endo, endocardium. (b) Network of free‐running Purkinje fibre strands comparable to the indicated structures in Figure 1e. (c) Zoomed‐in section of the free‐running Purkinje fibre network. (d) Endocardial section corresponding to box D in (a) Purkinje fibres (PF) are found sub‐endocardially surrounded by collagen and thin elastic fibres. CM, cardiomyocytes. (e) A midmyocardial section corresponding to box E in (a). The collagenous streaks are associated with vasculature, not with Purkinje fibres. A indicates an artery. (f) QT interval as a function of RR intervals (black symbols) from the ECG loggers placed in the freely roaming lions in Kgalagadi Transfrontier Park (*n* = 12). For comparison, the QT interval as function of the RR interval from the individual anaesthetized zoo lions is plotted in red. Error bars indicate the standard deviation.

The bundle of His divides into the left and right bundle branches that are found subendocardially in the septum, where they activate the ventricular walls (Figure 4a). The left ventricle of the lions all had remarkable networks of free‐running Purkinje strands (Figures 1e and 4d) in continuation with a subendocardial network of Purkinje strands. Compared to ventricular cardiomyocytes, the Purkinje‐fibre cells are short and ovoid myocytes embedded within a thick layer of connective tissue. Most of the collagen fibres are undulating or wavy and arranged parallel to the direction of the Purkinje strands. A network of thin elastic fibres embeds the collagen fibres. The Purkinje‐fibre cells contain myofibrils organized in parallel (Figure 4b). There is a varying degree of central clearing in the Purkinje‐fibre cells, possibly due to the washout of glycogen deposits during sample preparation. The ventricular, free‐running Purkinje strands consist primarily of Purkinje fibres, but connective tissue and cardiomyocytes are also present (Figure 4d, e).

The free‐running Purkinje network is likely important for rapid electrical activation of the papillary muscle of the left ventricle. No Purkinje fibres were found in the midmyocardium or epicardium. Collagen streaks are present in the midmyocardium and epicardium, but they are found only in association with the vasculature (Figure 4c).

### 3.4 Different QT intervals in wild and zoo‐kept lions

The QT interval on the ECG reflects the time from ventricular depolarization to the end of ventricular repolarization. In larger mammals, repolarization duration is heart‐rate dependent, and these data confirm that this is also the case for conscious lions (Figure 4f). The QT intervals in the conscious, wild lions were longer than the QT intervals measured in anaesthetized zoo‐kept lions (Figure 4f).

**FIGURE 5:**
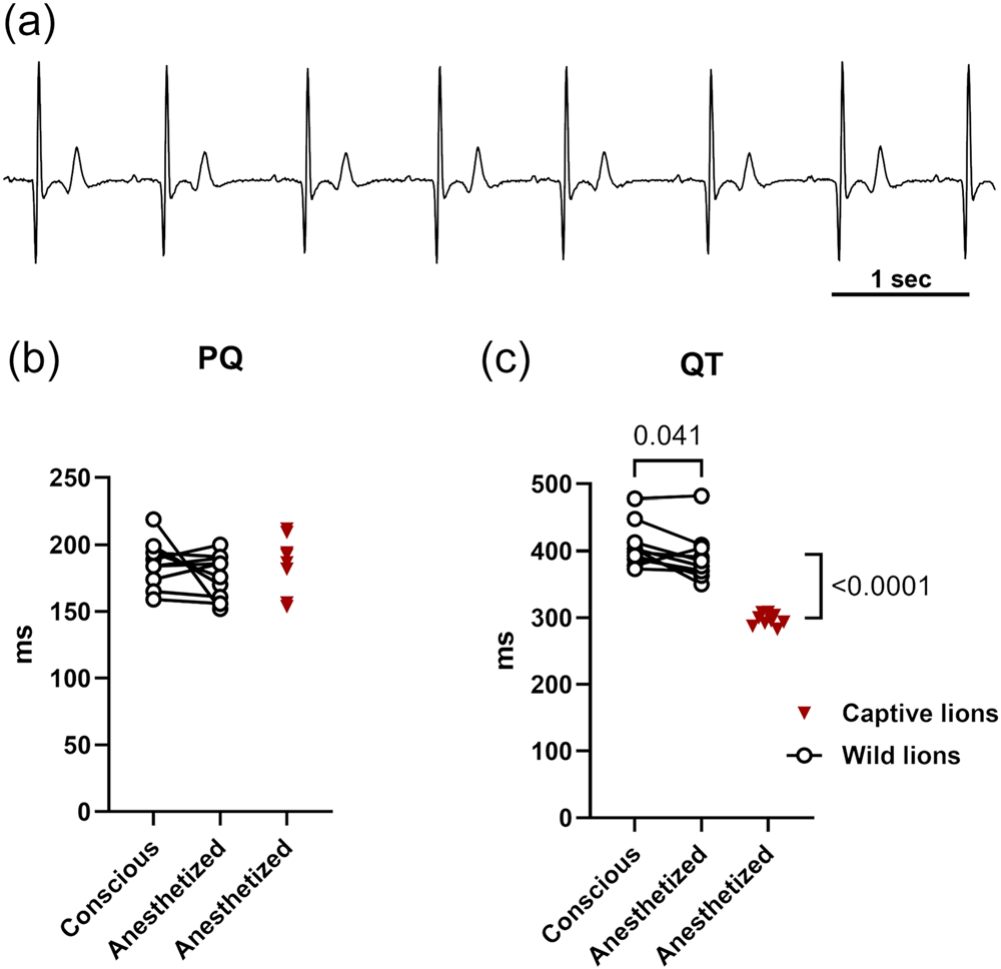
Shorter QT interval in the zoo‐kept lions. (a) Exemplar 7.5‐s ECG trace from a conscious, wild lion. The heart rate is 58 beats/min and the QT interval is 412 ms in this example. (b) PQ intervals from wild lions during conscious circumstances and during anaesthesia at matched heart rates (open circles, *n* = 12; Student's paired *t*‐test). For comparison, PQ intervals from the anaesthetized lions bred and housed in captivity is illustrated (red inverted triangles, *n* = 9; same data are illustrated in Figure 3d; Student's unpaired *t*‐test). No statistically significant differences were identified. (c) QT intervals from wild lions during conscious circumstances and during anaesthesia at matched heart rates (open circles, *n* = 12; *P*‐value from Student's paired *t*‐test). For comparison, QT intervals from the anaesthetized zoo‐kept lions in Denmark are illustrated (red inverted triangles, *n* = 9; same data are illustrated in Figure 4f; *P*‐value from Student's unpaired *t*‐test).

A representative ECG trace from a conscious lion is shown in Figure 5a. To gauge the effects of anaesthesia, we used the first few ECG‐recording epochs from the wild lions as representative examples from anaesthetized lions and compared these with ECGs at a comparable heart rate in the same animal during conscious conditions. The PQ interval did not change with anaesthesia (Figure 5b); however, the QT interval shortened from 420 ± 41 ms under conscious conditions to 398 ± 40 ms during anaesthesia (*P* = 0.0097; Figure 5c). Nevertheless, this anaesthesia‐induced shortening of the QT interval was not sufficient to reach the levels of the short QT intervals observed in the zoo lions prior to euthanasia. The heart rates in the two lion populations during anaesthesia were comparable; however, the QT intervals in the zoo‐kept lions were significantly shorter than in the wild lions (Figure 5c).

### 3.5 Detailed electrocardiographic analysis of anaesthetized lions

**FIGURE 6:**
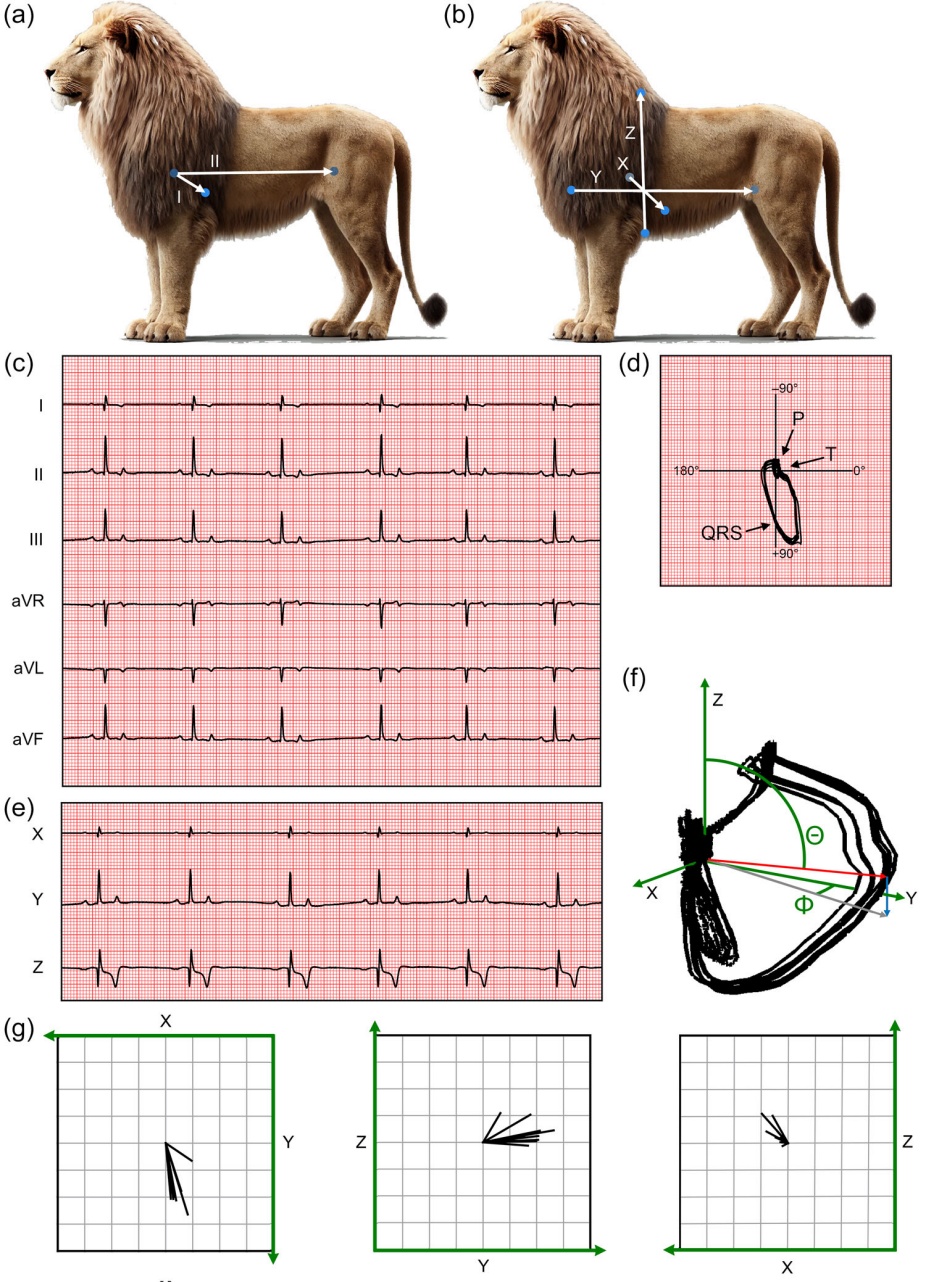
Electrical activity in the lion heart. (a) Position of electrodes in the Einthoven configuration. The vectors are directed from the negative electrode (right front limb) to the positive electrode (left front and left hind limb). A ground electrode (not shown) was placed on the right hind limb. (b) Position of electrodes in the orthogonal configuration. The vectors are directed from the negative electrode to the positive electrode. (c) Electrocardiogram obtained in the Einthoven configuration; 25 mm/s recording speed, 1 mV/cm amplitude. (d) 2D vector loops of the entire 7‐s recording shown in (c). Lead aVF (vertical) is plotted against lead I (horizontal); 0.5 mV/cm. (e) Electrocardiogram obtained in the 3D orthogonal configuration. (f) 3D vector loops based on ECG complexes recorded in the orthogonal configuration. The mean electrical axis is shown as a red arrow. The elevation, which is the angle between the mean electrical axis and the *Z*‐axis running in a ventral‐to‐dorsal direction, is indicated by Θ. The azimuth is the angle between the *X*‐axis and the projection of the mean electrical axis on the horizontal plane (grey arrow). For simplification, the angle indicated by Φ in the figure is between the *Y*‐axis and the projection, so the azimuth is 90 − Φ. (g) Projections of the mean electrical axis on the *XY*, *YZ* and *XZ* planes from all nine lions. All axes are 4 mV long and all vectors originate at (0, 0). The lion in Panel A and B was generated using publicly available deep‐learning algorithms that generate pictures.

We obtained two different ECGs from the nine anaesthetized zoo lions. These ECGs were recorded with more leads (Figure 6a, b), larger anatomical distance between the leads, and a higher temporal resolution than was possible using the data loggers in the conscious lions. A six‐lead ECG in the Einthoven configuration from a representative lion is shown in Figure 6c. In lead II, we observe positive P waves, upright QRS complexes with no S‐wave, and positive, narrow and symmetric T waves. We did not identify differences in electrophysiological parameters when comparing the obtained values from the three Danish zoos, and we have pooled the ECG data from a lead‐II analysis in Table 1. Compared to human data, the QRS duration and QT interval of the lion are shorter (Haarmark et al., 2010; Macfarlane, 2018). The vectorcardiogram is provided in Figure 6d and shows a large, narrow QRS loop with a cardiac axis of 83 ± 14°.

**TABLE 1:** Analysis of the ECG (lead II) in anaesthetized zoo‐kept lions.

RR interval	1099 ± 138 ms
PQ interval	185 ± 20 ms
QRS duration	62 ± 11 ms
QT interval	297 ± 9 ms
*n*	9

The mean ± standard deviation is shown. *n* indicates the number of biological replicates (nine lions). The ECG was recorded once per animal, that is, the number of technical replicates is 1.

Figure 6e shows *X*, *Y*, and *Z* lead recordings from the same lion as in Figure 6c. The large, positive amplitude of the QRS complex in lead *Y* indicates that the mean ventricular electrical axis is pointing towards the hind legs. The three‐dimensional vector cardiogram in Figure 6f depicts a flat, round QRS loop in the *YZ* plane. The T‐wave loop in the vectorcardiogram points in the negative *Z* direction. The maximal instantaneous vector is 0.99 ± 0.22 mV in amplitude and points largely in a caudal direction: the elevation (Θ), which is the angle from the *Z*‐axis running in a ventral‐dorsal direction, is 75 ± 23°; the azimuth (Φ), which is the angle between the *X*‐axis and the projection of the maximal vector on the horizontal plane, is 73 ± 16° (*n* = 9). Figure 6g shows the individual projections of the mean electrical axes in the horizontal (*XY*), sagittal (*YZ*) and transverse (*XZ*) planes from the nine lions.

**FIGURE 7:**
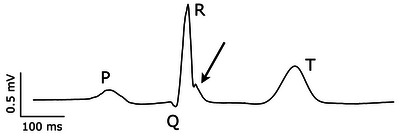
Early repolarization notch. Enlarged ECG trace from a lead II in an anaesthetized zoo lion. Electrical noise is removed from the signal by aligning complexes to the peak of the R wave and generating an averaged signal from 10–20 beats (Speerschneider & Thomsen, 2013). Note the prominent J wave (arrow) representing early repolarization of the ventricular myocytes and the short QT interval (330 ms). Additionally, P, Q, R and T waves are indicated on the trace.

We did not observe any supraventricular or ventricular arrhythmia in the recordings from the anaesthetized lions. Notwithstanding, the quality of the recordings allowed a detailed inspection of the ECG. We observed various degrees of notching on the descending part of the R wave in seven of nine lions (Figure 7), reminiscent of a J‐wave associated with an early repolarization syndrome.

## 4 DISCUSSION

Our study provides for two unique comparisons in lions. First, paired ECG recordings from conscious and anaesthetized African lions were assessed. Next, the ECGs from anaesthetized, wild lions were compared to those from anaesthetized zoo‐kept lions in Denmark. Moreover, we provide the first detailed histological description of the cardiac conduction system in lions and account for the three‐dimensional electrical axis.

Electrophysiological parameters have primarily been used for monitoring heart rate and rhythm and gauging the depth of anaesthesia in immobilized lions and during surgery. Larsson et al. (2008) described electrocardiographic recordings from 27 zoo lions under anaesthesia with xylazine and ketamine, and reported a mean heart rate of 64 ± 12 beats/min. There were positive P waves in lead II and predominantly positive QRS complexes with no S wave in lead II. The mean cardiac axis in the Einthoven plane was between 60 and 90° in all lions. QT intervals were 306 ± 24 ms with no significant differences between male and female lions in the measured electrophysiological parameters (Larsson et al., 2008). Omóbòwálé et al. (2017) obtained similar heart rates and QT intervals (66 ± 12 beats/min and 320 ± 35 ms, respectively) in zoo‐kept lions anaesthetized with a similar xylazine–ketamine combination. The mean cardiac axis in the Einthoven plane was 89 ± 5° (Omóbòwálé et al., 2017). Both studies investigated anaesthetized zoo lions.

To the best of our knowledge, this is the first report of ECGs from conscious lions. The data loggers revealed higher heart rates at night, in agreement with the nocturnal behaviour of these large apex predators (Wu et al., 2018). In addition to revealing the diurnal rhythms in heart rate (Figure 2d), the long recordings of the loggers also enabled determination of the PQ and QT intervals at a range of different heart rates (Figures 3d and 4f, respectively). The shorter recordings of the zoo‐bred lions kept in human care allowed for additional ECG leads and higher temporal resolution.

The paired comparison of ECGs from anaesthetized and conscious wild lions showed comparable PQ intervals (Figure 5b) and a small, but statistically significant, shortening of the QT intervals (Figure 5c) upon anaesthesia. Importantly, these comparisons were made at comparable heart rates. Medetomidine was included in the anaesthetic mix for both wild and zoo lions and is an α_2_‐adrenergic receptor agonist that is associated with vasoconstriction, delayed AV conduction and thus prolongation of the PQ interval (Schöndorfer et al., 2023). The remaining drugs (ketamine, midazolam, butorphanol, tiletamine and zolazepam) are not associated with altered AV conduction, nor with a change of the QT interval. It is at present unclear what mechanism underlies the small QT abbreviation in the wild lions.

More fascinating is the quite dramatic >100 ms difference in QT intervals between anaesthetized, wild African lions and the anaesthetized zoo lions bred in captivity (Figure 5c). Potential environmental or genetic differences may underlie this disparity in QT intervals. Miller et al. (2023) analysed microsatellite markers in wild and zoo lions in South Africa. They found that the genetic composition and diversity of the zoo lions are representative of those of wild lion populations in South Africa. Thus, it remains purely speculative whether a genetic mechanism may explain the short QT in the lions in the Danish zoos. Notwithstanding, the QT intervals from the anaesthetized, zoo‐kept lions (297 ± 9 ms; Table 1) appear to be in agreement with the previously reported measurements from anaesthetized zoo lions (306 ± 24 and 320 ± 35 ms, respectively; Larsson et al., 2008; Omóbòwálé et al., 2017).

Environmental differences between the Kgalagadi Transfrontier Park and the Danish Zoos are obvious, but the effects have not been studied in lions. Wild lions undoubtedly undertake more physical activity than their sedentary zoo counterparts. Human athletes have longer QT intervals than sedentary individuals, also, when the lower resting heart rate of the athlete is taken into account (Christou et al., 2022; Lengyel et al., 2011). Thus, it is possible that the shorter QT interval in the zoo lions is due to physical inactivity. Nutrition is more constant in the zoo lions; and their 24 h diurnal rhythms are presumed to be adjusted to align with the activities of the zoos (Seyrling et al., 2022). Although the wild lions were captured at night, the QT intervals in Figure 5c were all recorded during daytime, and thus at the same time as the ECG recordings in the zoo‐kept lions. Moreover, the amplitude of the diurnal rhythm of the QT interval (not shown) is 11.7 ms, and thus insufficient to account for the >100 ms difference. Finally, the difference in ECG morphology between the Einthoven limb leads (Figure 6c) and the recordings from the subcutaneous logger with a small inter‐electrode distance (Figure 5a) may contribute slightly to the observed differences.

The two‐ and three‐dimensional ECG recordings from the lions (Figure 6) are clearly different from those of the horse, which we published recently, using the same experimental set‐up (Elbrønd et al., 2023). The cardiac electrical axis in the two‐dimensional Einthoven leads is 83 ± 14° in the lions (*n* = 9) compared to −82 ± 22° in the horses (*n* = 11), that is, pointing in an almost opposite direction. This reflects the rS‐morphology (i.e., small amplitude R wave, large amplitude S wave) of lead II and aVF in the horse, compared to the large amplitude R wave in the same two leads in the lion. The peak of the three‐dimensional vectorcardiogram of the lion points in the caudal direction (both elevation and azimuth approaching 90°), which is approximately parallel to the anatomical axis of the lion's heart. The electrical and anatomical axes in the horse are pointed in opposite directions (Elbrønd et al., 2023; Hesselkilde et al., 2021). The larger amplitude of the electrical vector in the horse likely reflects its larger heart mass.

There is mounting evidence to suggest that a cardiac electrical axis from the heart's base to the apex is found in mammalian species with Purkinje fibres located uniquely on the endocardial surface of the heart. This requires a transmural activation that is governed by myocyte‐to‐myocytes interactions and is found in species like dogs (Hamlin & Smith, 1965), humans (Gómez‐Torres et al., 2021; Ohkawa, 2008) and lions (Figure 4). In contrast, an electrical axis in the opposite direction, from apex to base, is found in hearts with relatively thick ventricular walls. Here, transmural Purkinje fibres are necessary to rapidly conduct the depolarizing wavefront from the endocardium to the epicardium. We find this functionally and anatomically in the horse (Elbrønd et al., 2023; Gómez‐Torres et al., 2021) and goat (Hamlin & Smith, 1965; Ohkawa, 2008).

Body mass was significantly larger in male lions compared to females; however, we did not identify any sex differences in heart rate or QT intervals (data not shown). Notwithstanding, this study was most likely insufficiently powered to recognize sex differences in cardiac electrophysiology. It is evident from Figure 4f that QT intervals prolong when the RR intervals lengthen, that is, when heart rate slows. This is also reported in many large mammals, including in humans. When comparing QT intervals at different heart rates, it can therefore be useful to ‘correct’ QT intervals for heart rate. Numerous formulas to adjust QT intervals to a single heart rate have been suggested (Bazett HC, 1920; Fridericia LS, 1920; Sagie et al., 1992); however, none have been validated in lions. In the present study, we compared QT intervals (i) in conscious and anaesthetized wild lions, and (ii) in anaesthetized wild and zoo‐kept lions (Figure 5c), and both these comparisons were made at comparable heart rates. For these reasons, we did not adjust QT intervals to heart rate in the present study.

Larsson and colleagues report a broader heart‐rate range in their anaesthetized lions than we do (Larsson et al., 2008). Our analysis of their published tables shows that there is no correlation between heart rate and QT intervals, which agrees with our data from nine anaesthetized lions. Conversely, in the conscious lions, heart rate affects the QT interval, and we analyse QT intervals at heart rate range from 30 to 120 beats/min (Figure 4f). To the best of our knowledge, such RR–QT relationships have not been reported from any species of comparable body mass, except for humans. In a study recording 24 h ECGs from trained and untrained humans, heart rate ranged from 43 to 120 beats/min, and the corresponding QT intervals ranged from 500 to 300 ms (Genovesi et al., 2007). This is comparable to the range of values underlying Figure 4f.

Figure 7 shows a representative, high‐resolution ECG from a zoo lion. Seven of the nine zoo lions had comparable J waves or notches on the descending slope of the R wave. This reflects a gradient in the cellular membrane potential as a result of spatial dispersion in depolarization or repolarization. It is likely to be caused by an endocardial‐to‐epicardial gradient in the transient, outward potassium current, which is active early in the cardiac action potential, and predominantly expressed in the epicardium of the heart (Calloe et al., 2008). Other potential mechanisms for J waves are hypothermia and hypercalcemia, both of which are unlikely under the present circumstances.

A shorter‐than‐normal QT interval is associated with an increased risk of life‐threatening cardiac arrhythmia in humans (Gaita et al., 2003; Schimpf et al., 2005), suggesting that the zoo‐kept lions may be at increased risk of sudden cardiac death. Figure 4f shows that the QT intervals do not change with heart rate in the anaesthetized zoo‐kept lions, and it is most likely the short QT interval per se rather than the lack of heart‐rate dependency that is associated with a higher risk of cardiac arrhythmias. In a recent report on the causes of death in zoo‐kept African lions in North America, cardiovascular mortality is infrequent, but the authors argue that this is an understudied area of lion health. Figure 7 also shows a narrow, symmetrical T wave, which is associated with a *lower* risk of cardiac arrhythmias (Isaksen et al., 2021; Vicente et al., 2015). The T wave (and any other deflection from the ECG's isoelectric line) represents a gradient in the heart, where some cells are depolarized, others are repolarized. Such gradients are the source of ectopic beats and are critical for sustaining re‐entrant arrhythmias, like ventricular tachycardia. A narrow and symmetrical T wave suggests that the gradient is brief, which makes the risk of arrhythmias lower. Thus, it is tempting to speculate that a proarrhythmic short‐QT interval is balanced by a narrow and symmetrical T wave, resulting in a cardiovascular mortality rate that is comparable to that of wild lions.

In conclusion, this study provides a comprehensive description of the cardiac anatomy, histology and electrophysiology of wild lions and lions bred and housed in captivity. QT intervals were significantly shorter in zoo lions, suggesting functional discrepancies between the cardiac electrophysiology of wild and zoo‐kept lions, which potentially could be related to physical fitness. Our findings emphasize the plasticity of cardiac electrophysiology and may be of value when reintroducing endangered species into the wild and when managing lions in human care.

## AUTHOR CONTRIBUTIONS

Field work, Denmark: Bertelsen, Calloe, Elbrønd, Sandgreen, Scharling, Stagegaard, Thomsen, Vincenti and Wang. Field work, South Africa: Bell, Bennett, Bertelsen, Daly, Gunner, Hart, Marks, van Rooyen, Scantlebury and Wilson. Analyses: Calloe, Elbrønd, Isaksen, Scharling, Thomsen and Vincenti. Drafting the manuscript: Calloe, Scharling and Thomsen. All authors have critically reviewed the manuscript and approved the final version. They all agree to be accountable for all aspects of the work in ensuring that questions related to the accuracy or integrity of any part of the work are appropriately investigated and resolved. All persons designated as authors qualify for authorship, and all those who qualify for authorship are listed.

## CONFLICT OF INTEREST

None declared.

## Supporting information



Data for Figures 2d, 3d and 4f.

## Data Availability

Data behind Figures 2d, 3d and 4f are provided as Supporting information with this publication. The remaining data that support the findings of this study are available from the corresponding author upon reasonable request.
